# Effects of Novaluron Exposure on the Oviposition and Expression of Ovarian Development Related Genes in Silkworm, *Bombyx mori* (Lepidoptera: Bombycidae)

**DOI:** 10.3390/insects16010009

**Published:** 2024-12-27

**Authors:** Meng-Jiao Wang, En-Xi Chen, Yi-Lin Ji, Yi-Xuan Qian, Yu-Ming Zhang, Lin Zhu, Guo-Dong Zhao, He-Ying Qian

**Affiliations:** 1 Jiangsu Key Laboratory of Sericultural and Animal Biotechnology, School of Biotechnology, Jiangsu University of Science and Technology, Zhenjiang 212100, China; wangmengjiao001128@163.com (M.-J.W.); 212211803101@stu.just.edu.cn (E.-X.C.); 13912710146@163.com (Y.-L.J.); qianyx0915@163.com (Y.-X.Q.); chirszhang27@163.com (Y.-M.Z.); zl15680879232@163.com (L.Z.); 2 Key Laboratory of Silkworm and Mulberry Genetic Improvement, Ministry of Agriculture and Rural Affairs, Sericultural Scientific Research Center, Chinese Academy of Agricultural Sciences, Zhenjiang 212100, China

**Keywords:** silkworm, novaluron, oviposition, ovary development, hormone regulation

## Abstract

*Bombyx mori* (Lepidoptera: Bombycidae) is an important economic insect, which mainly feeds on mulberry leaves and is widely used in many research fields. The growth and development of silkworm larvae are easily affected by the use of chemical insecticides such as novaluron, a benzoylurea insecticide. However, the effect of novaluron exposure on the reproduction of silkworms has not yet been studied. In this study, the effect of trace novaluron on the oviposition of silkworms and histopathological changes were first evaluated, and then the gene expression level changes after novaluron exposure were also determined by employing qRT-PCR. It was found that the number of eggs and the hatching rate of eggs in silkworms decreased significantly after feeding on leaves with a trace amount of novaluron (*p* ≤ 0.01). Furthermore, novaluron exposure could affect the development of ovary tissue by reducing the number of oocytes and oogonia in the ovaries of silkworms fed with novaluron. In addition, the transcription levels of genes related to ovary development (*Vg*, *Ovo*, *Otu*, *Sxl-S* and *Sxl-L*) and hormone regulation (*EcR* and *JHBP*2) showed varying degrees of downregulation at 24 h, 48 h, and 72 h after novaluron treatment (*p* ≤ 0.05). Therefore, we speculated that novaluron can affect the energy metabolism, ovary development, and egg formation of silkworms, thus leading to reproductive disorders of silkworms after novaluron exposure.

## Simple Summary

Novaluron is a benzoylurea insecticide, which can inhibit the synthesis of chitin during the growth process of insects, affecting the growth and development of insects, and has high ovicidal activity. Mulberry leaves are the main food for silkworms. Feeding on mulberry leaves contaminated with pesticides can lead to acute or chronic poisoning of silkworms and damage various organs of larvae, which ultimately affect the development of silkworms and silk yield. This study investigated the effects of adding a trace of novaluron on the expression of genes related to reproduction and ovarian development in silkworms. It was found that novaluron exposure had a significant effect on silkworms, with a significant decrease in the number of eggs laid and the hatchability of eggs, and the morphological structure of the ovaries was altered as observed by hematoxylin-eosin staining, which showed uneven distribution of cells, a relative decrease in the number of cells, and the appearance of vacuoles. The transcriptional levels of genes related to ovarian development showed a downward trend under the treatment of novaluron. The experimental results showed that the novaluron caused poisoning of silkworms, interfered with the normal formation of ovaries, and affected the growth and development of silkworms.

## 1. Introduction

In China, sericulture is a historical classic industry with profound traditional culture, and it is also one of the few agricultural industries with industrial scale advantages in the world. *Bombyx mori* (Linnaeus, 1758) belongs to Lepidoptera, Bombycidae, and is an important economic and model insect [1]. Silkworms have great similarity with humans in genetic mode, basic material metabolism, and energy metabolism, which provides a more suitable and operable research model for some basic and applied disciplines of life science [2]. Silkworms are a kind of silk-producing insect that has important economic value and a detailed research background of molecular biology and genetics [3,4]. However, with the intensification of agricultural environment pollution, such as pesticides and abuse of feed additives, the normal growth and development of silkworms have been severely affected, so the silk industry has been facing a great threat [5].

Silkworms have been domesticated for a long time under excellent conditions suitable for growth and development, which makes silkworms sensitive to most insecticides. Silkworm poisoning due to inappropriate selection of pesticides or incorrect use of methods often occurs and has become one of the main bottlenecks in the development of sericulture [6]. Novaluron is a benzoylurea insecticide that can prevent chitin synthesis during the molting stage of an insect, preventing the formation of new epidermis and leading to insect death [7]. In addition, novaluron can also regulate the growth and development of insects and destroy the enzyme system, and it has high ovicidal activity [8]. Studies have shown that phoxim can regulate the expression of genes in the IIS pathway, resulting in slow growth of silkworms, damaging the peritrophic matrix, reducing the immune response of silkworms, and leading to susceptibility to and injury from foreign pathogens [9,10]. Body weight loss was observed after trace acetamiprid exposure; the ovaries and fallopian tubes were abnormally developed, and the egg production also decreased [11]. After exposure to NaF, significant reduction in cocoon quality, survival rate, fecundity, and hatchability were observed [12].

Silkworms belong to the complete metamorphosis of insects, with four stages, namely egg, larva, pupa, and moth [13]. Ovulation is not only a process of genetic information transmission and energy storage, but also a process related to morphogenesis [14]. The normal development of the ovaries is the prerequisite of ovulation, which has an important effect on egg production and silk yield [15]. Vitelline protein is the main component of the female yolk in all oviparous animals. It accumulates rapidly in the vitelline stage and is stored in the egg; it is the main nutrient source for the early development of the embryo and larva [16]. Vitellogenin (Vg) is a precursor of vitellin (Vn); it has the highest expression level in the fat of the body and can provide nutritional and functional substances such as amino acids, vitamins, and trace elements for embryonic development [17]. Ovo transcription factor is a family of proteins encoded by the *Ovo* gene, which has been found in *Drosophila*, silkworms, and mammals. Ovo protein plays a very important regulatory role in the growth and development process, and studies have shown that the Ovo protein plays a crucial role in ovarian development and sex determination in *Drosophila* [18]. Sex determination is the result of the evolutionary selection of species and the basic characteristics of insect life activities, which can affect all aspects of individual development, such as embryogenesis, sex differentiation, reproduction, and other physiological processes. Sxl is the regulatory center of sex determination and the hinge of germ cell differentiation [17,18].

The development of insects is mainly regulated by the synergistic effects of juvenile hormone (JH) and 20-hydroxyecdysone (20E) [19]. Juvenile hormone is a key hormone that regulates the life cycle of insects and is mainly responsible for maintaining larval growth and preventing larval metamorphosis towards pupae and moth [20]. 20-hydroxyecdysone mainly regulates larval molting and the metamorphosis development of larvae, pupae, and moths, and it can act on the insect epidermis, promote the formation of a new epidermis, and enhance the activity of chitinase [21]. As a gonadotropin, JH plays an important role in regulating the development, molting, and metamorphosis of silkworms [22,23]. Juvenile hormone binding protein (JHBP2) is the carrier of JH transport and function in the body and can form a complex with JH, which is transported to the target tissues such as the ovaries to play various roles [24]. 20E binds to the receptor EcR/USP and then regulates the target tissue by activating the transcription of the corresponding response genes [25].

In this study, we evaluated the effects of trace novaluron on the laying of eggs and gene expression in silkworms and clarified the response of the ovaries to novaluron exposure. The results of this study provided a theoretical basis for the study of the response mechanism of silkworms to novaluron exposure and provided a reference for the prevention and control of pesticide poisoning of silkworms in silkworm breeding areas.

## 2. Materials and Methods

### 2.1. Insect Strains

The silkworm variety P50 was selected and preserved by the Sericulture Scientific Research Center of the Chinese Academy of Agricultural Sciences (latitude: 119.37; longitude: 32.11). The 1st instar to 3rd instar larvae were fed with mulberry leaves at a temperature of 28 ± 1 °C and relative humidity of 85%, and the 4th instar to 5th instar larvae were fed with mulberry leaves at a temperature of 25 ± 1 °C and relative humidity of 70% and kept ventilated.

### 2.2. Chemicals

Novaluron (C_17_H_9_ClF_8_N_2_O_4_) was purchased from ADAMA Ltd. (Beijing, China), and in order to ensure the survival of the silkworms, we used a dose of novaluron (0.001 mg/L) below the minimum approved dose for field use. The working solution was prepared by diluting the stock solution with ddH_2_O to a final concentration of 0.001 mg/L. Fresh mulberry leaves were soaked in an aqueous solution of 0.001 mg/L novaluron and dried naturally after 1 min. The chemical reagents used for extracting total RNA and reverse transcription were purchased from Nanjing Vazyme Biotechnology Co., Ltd. (Nanjing, China), and the chemical reagents used in qRT-PCR were purchased from Beijing Total Gold Biotechnology Co., Ltd. (Beijing, China). 

### 2.3. Sample Preparation

The larvae were divided into 3 control groups and 3 treatment groups. There were 120 larvae in each group, of which 60 were used for statistical data and 60 were used for anatomical data. Larvae in the control group were fed with fresh mulberry leaves all the time, and silkworms in the treatment group were fed with fresh mulberry leaves until the 2nd day 5th instar, fed with novaluron-treated mulberry leaves once, and then continued to be fed with fresh mulberry leaves. At 24 h, 48 h, and 72 h after treatment, silkworms were randomly selected for dissection, the ovaries were isolated and washed with cold phosphate-buffered saline (PBS), and 3 biological replicates were established and stored in an ultra-low-temperature refrigerator at −80 °C. After the remaining silkworms emerged, healthy males and females were selected to mate together. After 6 h, the female and male moths were separated; the female moths laid eggs, and then the number of eggs was counted.

### 2.4. Histopathological Evaluation

At 96 h after novaluron exposure, ten ovaries were randomly collected from control and treatment groups, respectively, then fixed and preserved in 4% glutaraldehyde fixing solution and embedded in paraffin. Three biological replicates were set. A 5 µm slice was cut from the paraffin block, fixed on the slice, and placed in a 40 °C incubator. Sections were stained with hematoxylin-eosin and observed under an optical microscope.

### 2.5. Isolation of Total RNA and Reverse Transcription

Total RNA was extracted by FreeZol Reagent (Vazyme). Reverse transcription was then performed using the HiScript III 1st Strand cDNA Synthesis Kit (+gDNA wiper). The total RNA was quantified by calculating the ratios of A260/A280 and A260/A230. The samples were confirmed as having good integrity, and the ratios of A260/A280 were between 1.8 and 2.1.

### 2.6. Quantitative Real-Time PCR

The qRT-PCR primers were designed using Premier 6.0 software, and the primer sequences are shown in Table 1. The chemical reagents used in qRT-PCR were purchased from Accurate Biotechnology (Hunan) Co., Ltd. (Changsha, China). The ABI 7300 system (Applied Biosystems, Foster City, CA, USA) was used for qRT-PCR. PCR reactions were performed with three technical replicates, and data were presented as the means ± SEM (standard error) of three technical replicates. *Actin*3 gene was used as housekeeping gene. The transcript levels of the genes were calculated according to the 2^−ΔΔCt^ method.

### 2.7. Statistical Analysis

The results of this study were shown as the means ± standard error (SE) of three independent measurements. To determine the effect of novaluron exposure on cocooning and gene expression in the silk gland of *Bombyx mori*, one-way *T*-tests were performed by different concentrations and exposure times for comparisons between groups. Asterisks denote significant differences as compared with the control group, as indicated by * *p* ≤ 0.05 and ** *p* ≤ 0.01.

## 3. Results

### 3.1. Effect of Novaluron on Egg Laying of Silkworms

**Figure 1:**
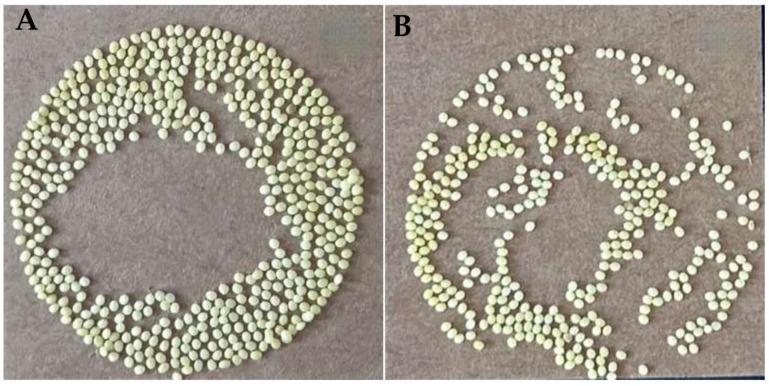
Effect of different treatments on laying silkworm eggs. (**A**) Control group; (**B**) Treatment group.

Mulberry leaves treated with 0.001 mg/L novaluron were fed to silkworm. The results of mating and laying eggs after cocooning, pupation, and emergence of the surviving silkworms are shown below. In the control group, the average number of eggs was 494, and the number of those hatching was 490, while in the treatment group, the average number of eggs laid was 344 (*p* ≤ 0.01), and the average number of hatching eggs was 275 (*p* ≤ 0.01); the hatching rate was reduced from 99.11% to 86.63% (*p* ≤ 0.01). In addition, novaluron also had an effect on the fertilization of silkworm eggs, and the results showed that novaluron could reduce the fertilization rate of silkworm eggs (Figure 1, Table 2).

### 3.2. Histopathological Analysis of Silkworm Ovaries

**Figure 2:**
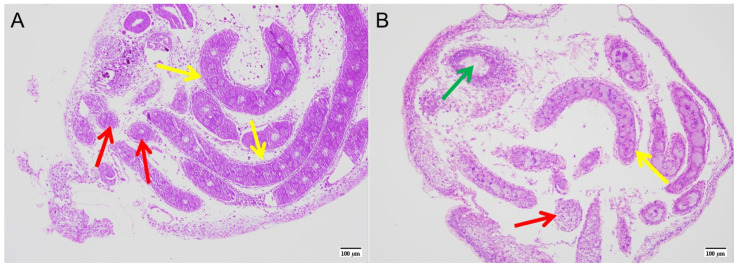
Histopathological images of silkworm ovaries after novaluron exposure. (**A**) Control group (100×); (**B**) Treatment group (100×). The red arrow indicates the oogonia, the yellow arrow indicates the oocyte, and the green arrow indicates the vacuole.

The ovaries were observed pathologically by hematoxylin-eosin staining and the paraffin embedded preparation technique. Tissue sections of the ovaries showed that the development of germ cells was significantly slower than that of the control group after exposure to a trace of novaluron. There was a marked decrease in the number of oocytes and oogonia, and some vacuoles appeared in the ovaries, while in the control group, more oocytes had already formed in the ovaries (Figure 2).

### 3.3. Effect of Novaluron Exposure on the Expression Level of Vg Gene in Ovaries

**Figure 3:**
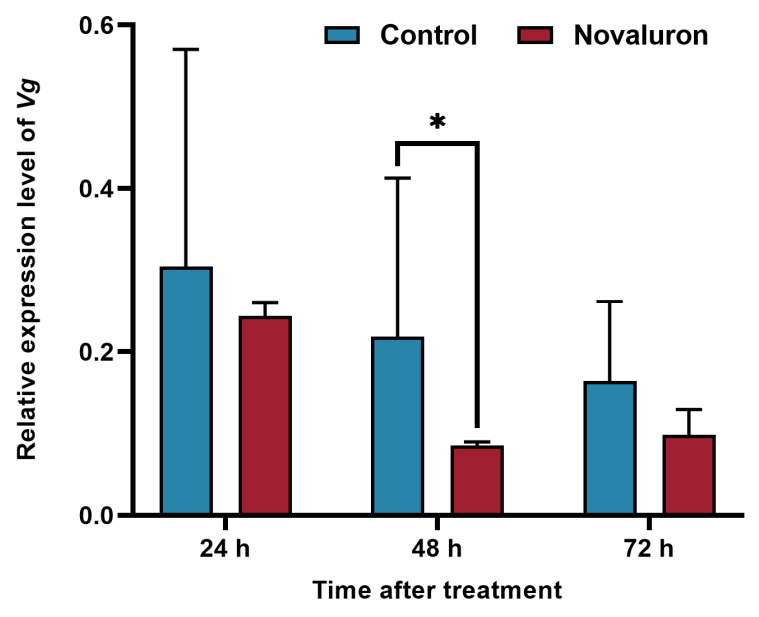
Effect of novaluron exposure on the expression level of the *Vg* gene in ovaries. Asterisks denote significant differences between treatments and controls, as determined using the pairwise *T*-test (* *p* ≤ 0.05). Values represent means ± SEM (*N* = 3).

In order to evaluate the effect of novaluron exposure on the Vg gene in the ovaries of silkworms, the expression of Vg in the ovaries of silkworms was measured by qRT-PCR, and the results were shown in Figure 3. Compared with the control group, the expression of Vg in the treatment group was downregulated to varying degrees at 24 h, 48 h, and 72 h, the downregulation was significant at 48 h, with a decrease of 39.06% (*p* ≤ 0.05), and the expression of the *Vg* gene in the ovaries of silkworms was inhibited by novaluron.

### 3.4. Effects of Novaluron Exposure on Expressions of Genes Related to Ovarian Development

**Figure 4:**
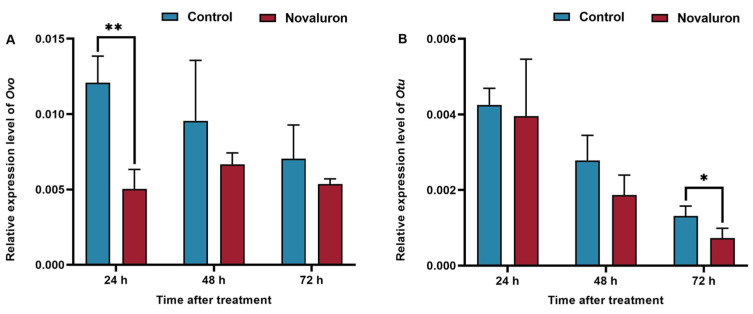
Effects of novaluron exposure on expressions of genes related to ovarian development. (**A**) *Ovo*; (**B**) *Otu*. Asterisks denote significant differences between treatments and controls, as determined using the pairwise *T*-test (* *p* ≤ 0.05, ** *p* ≤ 0.01). Values represent means ± SEM (*N* = 3).

To study the effect of novaluron exposure on genes related to ovarian development in silkworms, the transcriptional levels of *Ovo* and *Otu* genes were detected by qRT-PCR. As shown in Figure 4, the expression level of the *Ovo* gene in the treatment group was lower than that in the control group at 24 h, 48 h, and 72 h, and the downregulation was most significant at 24 h, which was 41.65% (*p* ≤ 0.01) of the control group. The transcription level of the *Otu* gene was lower than that of the control group at 24 h, 48 h, and 72 h, with 93.16%, 67.22%, and 55.28% (*p* ≤ 0.05), respectively.

### 3.5. Effects of Novaluron Exposure on the Transcription Levels of Genes Related to Sex Differentiation in the Ovary

**Figure 5:**
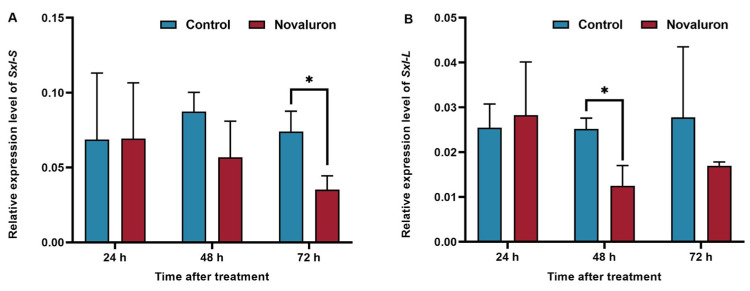
Effects of novaluron exposure on the transcription levels of genes related to sex differentiation in the ovary. (**A**) *Sxl-S*; (**B**) *Sxl-L*. Asterisks denote significant differences between treatments and controls, as determined using the pairwise *T*-test (* *p* ≤ 0.05). Values represent means ± SEM (*N* = 3).

Selective shearing of female-specific genes plays an important regulatory role in ovarian development. The results showed that the expression level of the *Sxl-S* gene was downregulated at 48 h and 72 h, with the most significant downregulation at 72 h, with 47.59% (*p* ≤ 0.05). The *Sxl-L* gene expression level was significantly lower than that of the control group at 48 h and 72 h; those values were 49.71% (*p* ≤ 0.05) and 61.21%, respectively (Figure 5).

### 3.6. Effects of Novaluron Exposure on Expressions of Genes Related to Hormone Regulation in Silkworms

**Figure 6:**
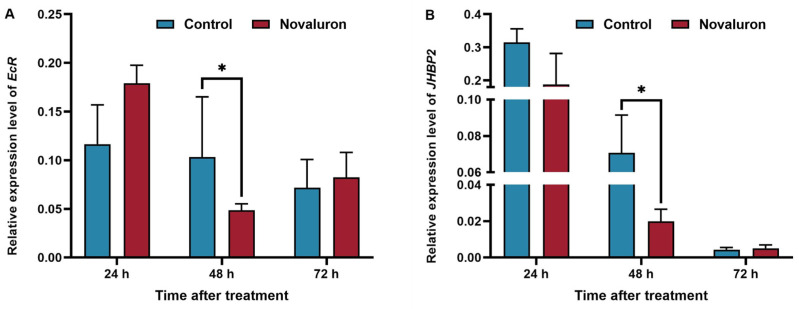
Effects of novaluron exposure on genes related to hormone regulation in silkworms. (**A**) *EcR*; (**B**) *JHBP2*. Asterisks denote significant differences between treatments and controls, as determined using the pairwise *T*-test (* *p* ≤ 0.05). Values represent means ± SEM (*N* = 3).

Juvenile hormone (JH) and 20-hydroxyecdysone (20E) play important roles in regulating the development, reproduction, and other life activities of silkworms. As shown in Figure 6, after exposure to novaluron, the expression of the *EcR* gene was significantly inhibited at 48 h, which was 47% of the control group. The expression level of the *JHBP2* gene at 24 h and 48 h was significantly lower than that of control group (59.82% and 28.22%, respectively, *p* ≤ 0.05).

## 4. Discussion

The domestication of silkworms has been ongoing for more than 5000 years, and many varieties with different biological characteristics have been formed under the long-term artificial directional selection [26]. Due to multiple factors such as unreasonable agricultural production layout, disjointed production schedule, and pesticide residues in the soil and water around mulberry orchards, mulberry leaves are polluted by pesticides, and mulberry worms which use mulberry leaves as their main feed are prone to acute or chronic poisoning [27]. In addition to the expression and regulation of a series of genes, external factors such as chemical pesticides, PH, temperature, and humidity can also affect the growth and development of silkworm ovaries. As a kind of benzoylurea insecticide that can prevent chitin synthesis, novaluron can regulate the growth and development of insects and destroy the enzyme system and has high ovicidal activity [8]. Generally speaking, novaluron mainly acts through ingestion and contact behavior, killing pests by preventing larvae from molting normally. Its pharmacological effect lasts for a relatively long time. Santorum et al. found that novaluron caused cell death in all ovarian organs of silkworm and significantly reduced egg production in female moths [28]. Novaluron also has toxic effects on the reproductive function of bumblebees, not only impairing the ability to lay eggs and the survival of eggs but also causing the death of most individual larvae [29]. One of the most destructive pests for potatoes is the Colorado potato beetle, and the fertility of the beetle was greatly reduced after novaluron treatment; meanwhile, the beetle’s eggs laid on the treated leaves did not hatch [30]. The results of this study showed that after the addition of novaluron, the number of eggs and the hatching rate of silkworms decreased, and the number of ovarian oocytes and oogonia in the treatment group decreased. The arrangement was loose, and vacuoles appeared, which means that novaluron destroyed the normal development of the ovaries of silkworms, seriously affected the egg laying and hatching, and leads to the obstruction of the reproductive development of silkworms.

The ovarian development of silkworms is not only affected by the external environment and exogenous chemicals but also closely related to the expression level of related functional genes. The eggs of silkworms are formed in the ovaries, and the amount of yolk protein produced during ovum formation plays a crucial role in embryogenesis and the provision of nutrients required for the life maintenance of the early larvae. Therefore, yolk protein is an essential nutrient for oocyte maturation and embryo development [31]. Silkworm yolk mainly consists of three proteins, namely vitellin (Vn), egg-specific protein (ESP), and 30-kDa proteins [32]. Vitellogenin (Vg), a precursor of Vn, is an important nutrient for egg formation and embryo development and is synthesized mainly in the female adipose bodies of oviparous animals [33]. Vg enters the ovary through vitellogenin receptor (VgR)-mediated endocytosis and is stored and utilized in the form of vitellogenin granules to provide nutrients and energy reserves for future embryo development [34]. In scanty vitellin mutation of silkworm, VgR cannot be separated from Vg and 30-kDa protein, and VgR cannot circulate, resulting in the lack of Vn and 30-kDa protein in eggs of vit mutants, leading to small eggs and white eggs of mutant silkworms and abnormal development of ovarian tubes after mutation of the *VgR* gene, so these findings suggested that Vg is essential for ovarian development [35]. RNA interferes with *BmVg* gene expression in female pupae, resulting in abnormal egg formation and embryonic development, suggesting that Vg plays an important role in this process [36]. In the present experiment, we found that the expression level of the *Vg* gene was downregulated after 24 h, 48 h, and 72 h of exposure to novaluron. It was hypothesized that due to the reduction in *Vg*, the yolk protein reduced correspondingly, resulting in the reduction of nutrients and energy in the development of the ovarian embryo, which interfered with the formation of the egg and the development of the embryo, and ultimately, the number of eggs and the hatchability rate decreased after exposure to novaluron.

Ovo transcription factors play a very important role in the regulation of energy metabolism in insects. Ovo-A and Ovo-B are only expressed in germ cells, with opposite regulatory activities, which can regulate ovarian development [37]. In ovarian cells, the *Ovo* gene can activate the ovarian tumor gene (*Otu*), and *Otu* gene expression is upregulated by *Ovo-B*, while *Ovo-A* inhibits *Otu* gene expression [38,39]. Previous studies have shown that the expression of the *BmOvo* gene can be interfered with by using RNA interference technology, and the results showed that when *BmOvo* gene is downregulated, the number of eggs is significantly reduced, accompanied by a decrease in the hatching rate. The *Otu* gene plays a variety of functions during germ cell formation in female *Drosophila melanogaster*, such as regulating the formation of fusion bodies and affecting the formation of actin bundles [40]. During the ovarian development of *Drosophila melanogaster*, the Ovo protein can bind to the promoter of Otu to regulate the expression of the *Otu* gene directly [38]. The mechanism of sex determination in the silkworm is important for physiological activities such as embryogenesis, sex differentiation, and reproduction. The Sex-lethal (*Sxl*) gene is the main switch gene for sex determination in *Drosophila* somatic cells and a key regulator of the sex determination network in the germ cells, which regulates the expression of the downstream *dsx* genes and is required for oogenesis [41]. The Sex-lethal gene can be selectively sheared, and previous studies have shown that the *Sxl-S* and *Sxl-L* genes play an important role in the regulation of ovarian development [42,43]. In this study, the expression levels of genes related to ovarian development were detected, and results showed that the expression levels of the *Ovo* and *Otu* genes in the treatment group were lower than those in the control group after treatment with novaluron, and the expression levels of the *Sxl-S* and *Sxl-L* genes reached their lowest levels at 72 h and 48 h, respectively. Therefore, we hypothesized that novaluron could cause abnormal shear regulation of silkworms. It disrupted the normal process of egg formation and eventually led to abnormal ovarian development.

Insect hormones are secreted by glands and can be transported to various parts of the body. The growth and development of insects are mainly regulated by the synergistic regulation of JH and 20E. Studies have shown that ovarian development is generally induced by 20E after the molt of larva [44]. 20E binds to its receptor EcR/USP to regulate target tissues by activating a series of primary and secondary response genes, which plays an important role in the transmission of the 20E signal [44]. The combination of EcR/USP can activate downstream gene expression, induce Vg synthesis, and provide nutrition for developing ovaries. Juvenile hormone was first discovered in the study of *Rhodnius prolixus* and can regulate a variety of physiological functions, such as metabolism, diapause, and immunity, in the adult stage of insects, especially in the development and maturation of female eggs [45,46]. In insects, as a type of carrier protein, JHBP combined with JH to form a complex, which transported JH to the target organs to regulate the development of insects and prevent JH from being degraded by non-specific enzymes in hemolymph [47,48]. This study’s results showed that the expression level of EcR in the treatment group was lower than that in the control group at 48 h after feeding, and the transcription level of the *JHBP2* gene was also significantly lower than that in the control group at 24 h and 48 h. Therefore, we speculated that the hormone metabolism in silkworms was affected by novaluron exposure through affecting the expression of *EcR* and *JHBP2*, resulting in the inhibition of the reproductive development of silkworms and reproductive disorders.

## 5. Conclusions

This study analyzed the effects of novaluron exposure on ovarian damage and ovarian development-related gene expressions in silkworms and elucidated the mechanism of novaluron-induced reproductive disorders in silkworms. After exposure to trace novaluron (0.001 mg/L), the morphological structure of silkworm ovaries changed, and the number of eggs and hatching rate decreased. In addition, novaluron inhibited the expression of genes related to ovarian development in silkworms (*Vg*, *Ovo*, *Otu*, *Sxl-S*, and *Sxl-L*), affecting the normal synergistic regulation of JH and 20E in silkworm larvae. Novaluron exposure can cause silkworm poisoning through regulating the expression level changes of related genes, and then the absorption and metabolism of nutrients were affected, interfering with normal ovarian formation and resulting in reproductive disorders in silkworms.

## Figures and Tables

**Table 1:** Primer sequences for qRT-PCR.

Gene Name	Primer Sequence (5′–3′)
Actin3	F: CGGCTACTCGTTCACTACC
	R: AGCAATTCACACAAGGCAGT
Vg	F: CTGCAACGCAAGGAAACCAA
	R: TGGCCGTACTTGAAGTGCAT
Ovo	F: GCAGCTGCTTTAGGACTACCAG
	R: CGTTAGCTTCAGTCGCCAAA
Otu	F: AACCACAACGCTGACCAGAA
	R: GTGGCCCTTGTTCTGATGGT
Sxl-S	F: CGCGTTACCTATTTAACATTTCGTG
	R: CCTCGGTACTGCTGTTGGAT
Sxl-L	F: CGGGATACTTGTTTGGTGGC
	R: AGACATGCTGCCCCAGTATC
EcR	F: TGATGGAGCAGAACAGGCAG
	R: CCTCTTCATCCGACTGCGTT
JHBP2	F: CAATGCCTTAGCAGTGCGAC
	R: TGAAGCGTATCACGACTCCC

**Table 2:** Effect of novaluron exposure on reproduction of silkworms.

	Control	Novaluron
Average Number of Eggs (Grain)	494 ± 18	344 ± 17 **
Average Number of Hatching Eggs (Grain)	490 ± 19	275 ± 11 **
Hatching Rate (%)	99.11 ± 0.35	86.63 ± 1.64 **
Percentage of Fertilized Eggs (%)	99.92 ± 0.11	88.54 ± 1.39 **

The experiments were repeated three times. The significance of differences is indicated by ** *p* ≤ 0.01.

## Data Availability

The original contributions presented in this study are included in the article. Further inquiries can be directed to the corresponding authors.
